# Expression pattern of endogenous PAR-4A & C after CRISPR/Cas9-mediated genome editing

**DOI:** 10.17912/micropub.biology.000075

**Published:** 2018-11-13

**Authors:** Vincent Roy, Olivier Gagné, Karim Hamiche, Jean-Claude Labbé, Patrick Narbonne

**Affiliations:** 1 Département de Biologie Moléculaire, de Biochimie Médicale et de Pathologie, Faculté de Médecine, Université Laval, Québec, Canada.; 2 Département de Biologie Médicale, Université du Québec à Trois-Rivières, Trois-Rivières, Canada.; 3 Département de Pathologie et Biologie Cellulaire, Institut de Recherche en Immunologie et en Cancérologie (IRIC), Université de Montréal, Montréal, Canada.

**Figure 1. mNG::PAR-4A & C are ubiquitously expressed, excluded from nuclei and enriched at the cell cortex.**
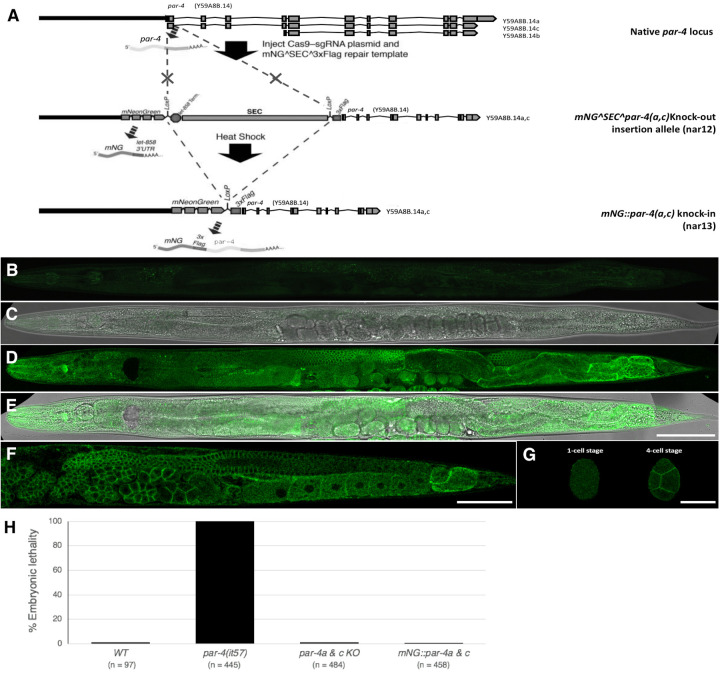
**(A)** Schematic depiction of *par-4* locus organization and the predicted transcripts from this gene before editing (top), after homologous recombination (middle), and after SEC removal (bottom). Adapted from (Dickinson *et al.* 2015). **(B-E)** Fluorescence micrographs (B, D) and pseudo-DIC/fluorescence-merge micrographs (C, E) of wild-type (N2) animals and *mNG::par-4a & c* animals taken with the same settings, 24 hours (25°C) after the late L4 stage. **(F)** Fluorescence micrograph showing a close-up of the germ line of a *mNG::par-4a & c* animal. **(G)** Fluorescence micrographs of 1- and 4-cell *mNG::par-4a & c* embryos. Scale bars: B-E, 80 mM; F, 55 mM; G, 27 mM. **(H)** Maternal effect embryonic lethality of the indicated genotypes at 25°C.

## Description

PAR-4/LKB1 is maternally expressed and required for the asymmetrical distribution of early embryonic determinants and viability in *C. elegans* (Morton *et al.* 1992; Watts *et al.* 2000; Tenlen *et al.* 2008). It is also implicated in a variety of postembryonic processes, including germline stem cell quiescence (Narbonne and Roy 2006; Narbonne *et al.* 2017), neuronal growth and polarity (Kim *et al.* 2010; Teichmann and Shen 2011), cytoskeletal rearrangements (Narbonne *et al.* 2010; Chartier *et al.* 2011), and metabolism (Narbonne and Roy 2009). Its expression pattern and subcellular localization has been determined in fixed animals by antibody staining (Watts *et al.* 2000). Here, we used CRISPR/Cas9-mediated genome editing to fluorescently label the two longest endogenous PAR-4 isoforms, PAR-4A and PAR-4C, with monomeric Neon Green (mNG) (Shaner *et al.* 2013), using the self-excising cassette (SEC) system (Dickinson *et al.* 2015) (Fig. 1A). We found ubiquitous *mNG::par-4a* & *c* expression and cytoplasmic and cortical enrichment of mNG::PAR-4A & C proteins in the germ line and early embryos (Fig. 1B-H), as previously described (Watts *et al.* 2000). Interestingly, we find that mNG::PAR-4A & C cortical enrichment is transiently lost in the pachytene area of the germ line (Fig. 1F), although it remains unclear whether this is functionally relevant.

The intermediate strain that still contains the SEC (Dickinson *et al.* 2015) is predicted to be null for *par-4a* & *c*, potentially leaving the shorter *par-4b* isoform functional. To evaluate the requirement for *par-4a* & *c* we examined the embryonic lethality (at 25°C) of the generated SEC-containing and SEC-excised strains. We found that the self-progeny of homozygous SEC-containing (*e.g.* *par-4a* & *c* null) animals is viable (Fig. 1H). This suggests that *par-4b* alone is sufficient to establish embryonic polarity and sustain the essential function of *par-4*. Consistent with this, to our knowledge, all existing *par-4* alleles that impair embryonic development disrupt *par-4b* (Morton *et al.* 1992; Watts *et al.* 2000).

## Reagents

Nematodes were cultured on standard NGM plates with *E. coli* (OP50) and maintained at 15 °C unless otherwise specified. N2 (Bristol) was used as wild-type. The following strains were also used: KK300: *par-4(it57)V*, UTR43: *par-4(nar12[Ppar-4a::mNG + loxP sqt-1(gf) Hygromycin (+) loxP 3X FLAG])V*, UTR45: *par-4(nar13[mNG :: par-4a & c])V*. UTR43 and UTR45 will be made available through the CGC.

pDD162 was modified as described (Paix *et al.* 2014) to generate two sgRNAs using the following primers (all 5’->3’): fwd Q5 1 gtgctcccgaggatgtcgagttttagagctagaaatagcaagt and fwd Q5 2 atgctccgtcgacatcctcgttttagagctagaaatagcaagt. pDD268 was modified as described (Dickinson *et al.* 2015) to generate the N-terminal mNG::SEC repair template using the following primers: 5’ arm *par-4* fwd acgttgtaaaacgacggccagtcgccggcaatttggtcgtttttggggtt, 5’ arm *par-4* rev catgttgtcctcctctcccttggagaccattgaagagagctctgaaattttt, 3’ arm *par-4* fwd cgtgattacaaggatgacgatgacaagagaatggacgcaccgtcaacttcatcaggagcacaaagcaaacttctg, and 3’ arm *par-4* rev tcacacaggaaacagctatgaccatgttatttccgaaaattgaacgattttt. Modified vector DNA sequences were confirmed by Sanger sequencing. The two sgRNA guide vectors were microinjected together with the repair template in wild-type animals that had been subjected to *cku-80(RNAi)* (Ward 2015) and a single *par-4* CRISPRed line was obtained; the SEC was excised as described (Dickinson *et al.* 2015).

All images were acquired from paralyzed live animals (0.1% W/V tetramizole in M9) with a Leica SP8 confocal microscope using the same parameters and were processed identically. Whole animals were stitched (Preibisch *et al.* 2009) and straightened using ImageJ.
